# The 1918 influenza pandemic: Ecological, historical, and evolutionary perspectives

**DOI:** 10.1093/emph/eoy021

**Published:** 2018-08-06

**Authors:** Charles L Nunn

**Affiliations:** Department of Evolutionary Anthropology and Duke Global Health, Director Triangle Center for Evolutionary Medicine (TriCEM), Duke University, Durham, NC, USA

One hundred years ago, a pandemic of unprecedented size and scope afflicted the human population. This pandemic was caused by an H1N1 influenza virus of avian origin. Over the course of 1918–1919, this virus infected approximately one-third of the nearly 2 billion people living on Earth at the time, resulting in at least 50 million deaths. The pandemic caused massive economic disruptions, severely strained many of the public health infrastructures that were designed to contain disease, and spread to the farthest and most isolated corners of the globe, often with especially deadly consequences in remote areas. The disease caused by this virus had several unusual characteristics that remain incompletely understood, including high mortality in previously healthy, young adults (i.e., 20–40 years of age). Questions also remain about the geographic origin of the pandemic, and the underlying drivers that led to emergence from avian hosts. The 1918 influenza pandemic remains one of the most feared epidemics in world history, and a rallying cry for more effective preparedness for future outbreaks of influenza and other viruses. It is well worth reflecting on this pandemic, including with an evolutionary lens.

In this special collection of papers, we present new evolutionary perspectives on the history of the 1918 influenza pandemic. Our collection includes a review by Dr. Margaret Humphreys that weaves together historical and evolutionary perspectives, providing a broad overview of the pandemic for those interested in evolutionary medicine. Dr. Michael Worobey and colleagues provide new perspectives on the geographic origins and timing of the first cases of the 1918 influenza pandemic, including insights from phylogenetic analyses and the causes of young adult mortality. In the time since the 1918 pandemic, a greater appreciation for the cross-species spread of viruses has emerged. To provide some of these new insights from a One Health perspective, Dr. Emily Bailey and her colleagues review new knowledge on influenza viruses. Lastly, as a provocative and forward-looking Commentary, Dr. David Fedson provides new insights to the causes of young adult mortality and proposes how this knowledge can be used to reduce mortality in future pandemics. He also outlines approaches for future testing of these proposals. In addition to these four papers, we have other papers that are in the works for this collection over the next year, and we welcome additional submissions.

As Editor-in-Chief of *Evolution, Medicine and Public Health*, I am excited to help orchestrate this highly interdisciplinary collection of papers. I would like to see other topical collections emerge on the pages of the journal, especially those that present different viewpoints based on rigorous science that move evolutionary medicine research forward. If you have ideas for other collections—including sets of papers from organized symposia—please contact me about the possibilities for publishing those papers at EMPH. 

**Conflict of interest**: None declared.

